# Stakeholders Perspectives on the Introduction of an Additional Injectable Vaccine Under the Universal Immunization Programme in India

**DOI:** 10.3390/vaccines13030334

**Published:** 2025-03-20

**Authors:** Pawan Kumar, Rashmi Mehra, Arindam Ray, Amrita Kumari, Kapil Singh, Rhythm Hora, Amanjot Kaur, Seema S. Koshal, Syed F. Quadri, Shyam Kumar Singh, Abida Sultana, Arup Deb Roy

**Affiliations:** 1 Immunization Division, Ministry of Health and Family Welfare, Government of India, New Delhi 110001, Delhi, Indiakksingh@unicef.org (K.S.); 2 John Snow India, New Delhi 110070, Delhi, India; rhythm_hora@in.jsi.com (R.H.); seema_koshal@in.jsi.com (S.S.K.); shyam_singh@in.jsi.com (S.K.S.);; 3 Bill and Melinda Gates Foundation, New Delhi 110067, Delhi, India

**Keywords:** immunization programs, vaccination, caregivers, health workers, injections

## Abstract

Introduction: In 2023, India’s National Technical Advisory Group on Immunization (NTAGI) recommended the inclusion of typhoid conjugate vaccine (TCV) in the Universal Immunization Programme (UIP). However, introducing TCV, an additional injectable vaccine (AIV), will potentially increase the number of injections administered in a single visit to a maximum of five (if given at the 9 to 12 months touchpoint) or four (if given in the second year of life). In this context, the present study aimed to explore the perspectives of program managers, service providers, and caregivers regarding introduction of an AIV in a single visit under the UIP. Methods: A mixed-method study was undertaken wherein quantitative data was collected by telephonic surveys, and qualitative data by key informant interviews (KIIs) and focus group discussions (FGDs). Purposive sampling encompassed eight states, eight districts, eight planning units, and 32 session sites. The qualitative data were thematically analyzed manually using Excel, while the quantitative data was analyzed using STATA 17. Results: A total of 1140 telephonic surveys, 96 KIIs, and 16 FGDs were conducted. The study revealed that program managers mentioned maternal emotional reactions as a significant concern and backed AIV introduction at the 9–12 months touchpoint. Vaccinators and community mobilizers favored the 16–24 months window with combined presentations and mentioned single-dose vials as the preferred approach for vaccine delivery. Caregivers acknowledged the benefits of vaccination but expressed discomfort and fear regarding multiple injections to the child in a single visit. Caregivers expressed a preference for a combination vaccine. No preference was reported among caregivers for the introduction of AIV to the 9–12 or 16–24 months touchpoints. Conclusion: Stakeholders’ perspectives on introducing an additional injectable vaccine in a single visit under the UIP are diverse. These will be helpful in developing an effective strategy for the future introduction of AIV under UIP.

## 1. Introduction

The Universal Immunization Programme (UIP) of the Ministry of Health and Family Welfare (MoHFW), Government of India (GoI) is one of the largest in the world in terms of the number of beneficiaries reached, the number of immunization sessions organized, and the geographical spread and diversity of areas covered [1]. In recent years, several new vaccines have become part of the UIP, which can be attributed to the success of the UIP in providing improved coverage to India’s population against vaccine-preventable diseases [2]. An additional injectable vaccine, however, would also imply an increased number of injections at a single vaccination visit, which further highlights concerns about infant pain, potential or perceived adverse effects, and uncertainty about vaccine effectiveness and interactions [3]. Available evidence suggests that there are key challenges to new vaccine introduction, distribution, and uptake. Therefore, there is a need to devise contextually appropriate strategies to minimize these challenges [4].

In 2023, the National Technical Advisory Group on Immunization (NTAGI) recommended the introduction of the typhoid conjugate vaccine (TCV) under UIP [5]. This was in line with the recommendation by the World Health Organization (WHO) that TCV should be introduced in the routine immunization (RI) program any time after 6 months of age [6]. Considering the existing bouquet of vaccines available under UIP, it was imperative to take cognizance of the potential implications of adding another injectable vaccine to the schedule. There exist two touchpoints after 6 months of age for the beneficiary under the UIP-, 9 to 12 months and 16 to 24 months, both of which currently have 3–4 injectable vaccines scheduled (as detailed in Table 1) [7]. No previous study has provided evidence suggesting the most acceptable/operationally feasible window to introduce an additional injectable vaccine (after taking into consideration the timelines suggested for introduction on the basis of immunogenicity, safety, efficacy, etc.). Also, no previous study has been conducted to understand the perspectives of key stakeholders with respect to the introduction of an additional injectable vaccine under the existing national immunization schedule.

Therefore, a study was conducted to understand the perspectives of caregivers, service providers, and program managers regarding introducing an additional injectable vaccine (TCV) under the UIP. The objectives of the study were as follows:

1. Explore service providers’ perspectives on including an additional injectable vaccine in a single visit under the UIP in the identified geographies (timeframe, ideal presentation, and preparedness).
2. Understand caregiver perspectives on the acceptability and/or unacceptability of including an additional injectable vaccine in a single visit (timeframe, challenges, concerns, and facilitators).
3. Preparedness measures: interventions that could improve the uptake of multiple injectable vaccines in a single visit under the UIP and their programmatic implications (i.e., systems preparedness, community engagement).

## 2. Materials and Methods

### 2.1. Research Design

We conducted a mixed-methods study to gather the perspectives of stakeholders including caregivers and service providers. The qualitative approach included key informant interviews (KIIs), which included interviews with state immunization officers (SIO), district immunization officers (DIO), medical officers (MOs), community mobilizers (accredited social health activist (ASHA workers)), and vaccinators (auxiliary nurses and midwives (ANM)) and focus group discussions (FGDs) with caregivers and parents (predominantly mothers) of children under the age of 2 years present at the session sites. For the quantitative data collection, a telephonic survey among caregivers using a structured questionnaire was utilized.

### 2.2. Data Collection

**Figure 1:**
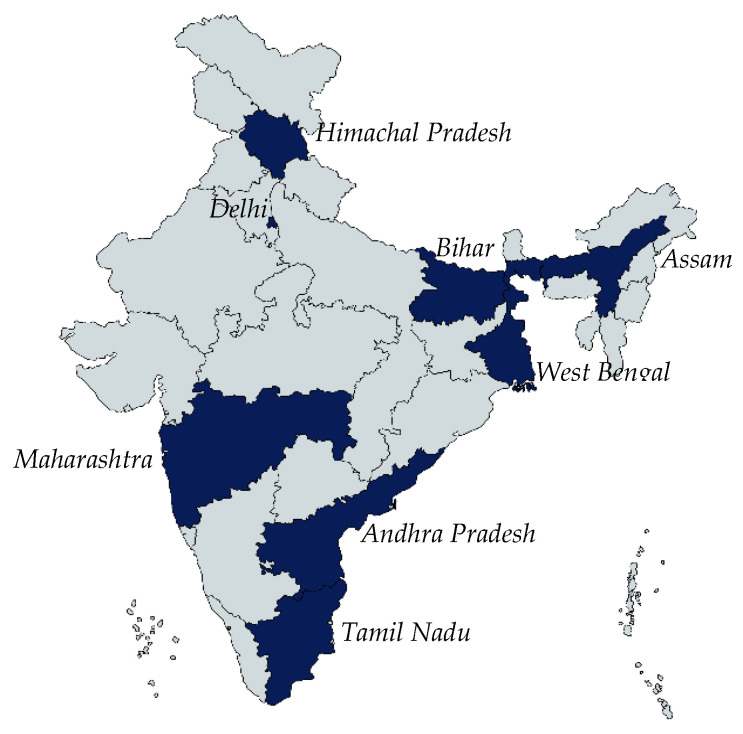
States covered in the present study.

For this study, states with existing typhoid surveillance sites, including Andhra Pradesh, Assam, Bihar, Himachal Pradesh, Maharashtra, Delhi, Tamil Nadu, and West Bengal (Figure 1), were included in the sample. In each state, one district was purposely sampled. In each district, two health facilities were randomly selected, considering the operational feasibility of conducting this study and the recommendation of the district officials.

For this study, we conducted 96 key informant interviews (KIIs) and 16 focus group discussions (FGDs), ensuring representation across key stakeholders (program managers, service providers, and caregivers). The selection of sample size was guided by existing qualitative research principles, which suggest that saturation typically occurs within 12–20 interviews per stakeholder group, depending on topic complexity. Thematic saturation was reached during data collection, confirming that the chosen sample size was sufficient for capturing diverse perspectives [8]. Additionally, triangulation with quantitative survey data strengthened the reliability of our findings.

The quantitative data collection was conducted as a telephonic survey using a structured, closed-ended questionnaire among male and female caregivers from the catchment area of the selected routine immunization session sites. The sample size for the telephonic survey was calculated separately for each state using the indicator—the percentage of full immunization coverage of children between 12 and 23 months of age and the projected population of women in the reproductive age group [9]. The total sample size of the study was estimated to be 1024 caregivers.

As a first step to enrolling participants in the survey, the ASHA supervisor and coordinator contacted caregivers individually to share information on the study, obtain their consent to participate, and share their phone numbers with the study team.

From this list, investigators randomly selected the phone numbers of caregivers and proceeded with the interview. Calls were scheduled between regular working hours, i.e., between 9:30 a.m. and 5:30 p.m. However, flexibility in call timing was considered if the caregiver asked to call at other convenient times, as per their availability. A maximum of three attempts were made to reschedule a call in the case that the respondent asked to call again on a specific date and time.

### 2.3. Data Analysis

The analysis of the quantitative data was undertaken as a descriptive analysis using STATA 17.

Qualitative data analysis included thematic analysis using the framework method, which involved the following steps: familiarization with the data; identifying a thematic framework; indexing; charting; mapping; and interpretation. The data were analyzed manually in Excel. The analysis using the framework analysis method was both deductive and inductive.

The data analysis was carried out in two stages. First, the quantitative and qualitative data were analyzed separately to understand stakeholder perceptions of introducing an additional injectable vaccine in a single visit under UIP. Next, we performed a comparative analysis to ascertain the common themes and patterns emerging from qualitative and quantitative data.

We followed a triangulation approach, where both qualitative and quantitative findings were analyzed separately and then compared to identify convergences, divergences, and complementary insights.

### 2.4. Ethical Considerations

Ethical approval for this study was obtained from Sigma-Institutional Review Board (IRB Number: 10024/IRB/23-24, Approval date: 12 July 2023). During the telephonic survey with caregivers, verbal consent was obtained over the phone before initiating the survey. The call was recorded only after obtaining audio recording consent from the respondent. The caregivers who did not consent to the survey after being contacted by the investigators were not interviewed and were replaced by the next caregiver on the list provided by the ASHA coordinator. Key informant interviews/FGDs were conducted in person. Written informed consent was obtained before starting the interview. Informed consent was obtained prior to data collection from the respondents of the IDIs and FGDs.

## 3. Results

A total of 1024 surveys were completed for quantitative data collection. The distribution of the quantitative data collection is elaborated in Table 2.

**Figure 2:**
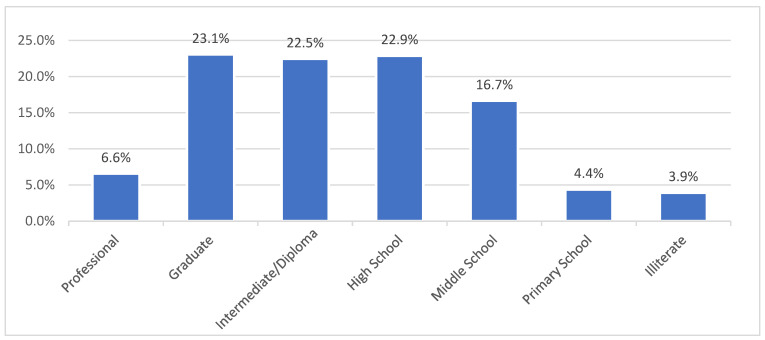
Education level of the respondents as per modified Kuppuswami socio-economic status scale (2024) [10].

The respondents were 85% women and 15% men. Of the total women respondents, 75% were housewives. The average age of the child was around 10.92 months with a standard deviation of 6.53 months. About 51.5% of caregivers reported having one child while 39.6% had two children. Around 4% of the respondents had no education, 16.7% had studied up to middle school, 45% of the sample finished high school or had a diploma, and 29.7% of the sample was graduate and above (Figure 2).

The qualitative data were collected from the selected eight states, with one district per state, followed by two health facilities per district. The qualitative sample included 96 KIIs with ANMs (n = 32), ASHAs (n = 32) and program managers (SIO (n = 8), DIO (n = 8), and MO (n = 16)). Additionally, 16 FGDs were conducted with caregivers across eight states. The caregivers were parents who had infants in the age group of 6–24 months (Table 3).

### 3.1. Stakeholder Perspectives

**Figure 3:**
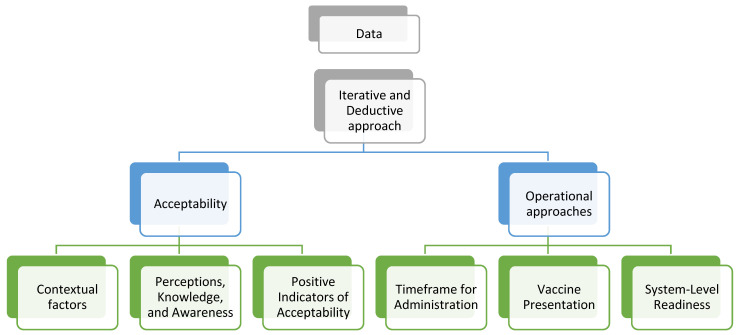
Theoretical framework utilized for qualitative data analysis.

The stakeholders’ perspectives collected through quantitative and qualitative data collection were analyzed through an iterative process. As in a previous study conducted in South Africa, themes that came up during the thematic coding and grouping of the qualitative data were used to develop the following theoretical framework that was utilized for analysis of the data [11] (Figure 3).

#### Acceptability

The acceptability of introducing an additional injectable vaccine under the Universal Immunization Program (UIP) was analyzed through stakeholders’ perspectives, segmented by themes such as contextual factors, perceptions, and positive indicators of acceptability. The insights reveal varying enablers and challenges across different stakeholders—program managers, service providers, and caregivers.

### 3.2. Contextual Factors

#### 3.2.1. Geography and Mobility Challenges

Difficult terrain, such as hilly areas and regions with poor access, significantly hindered vaccine accessibility. The commute to PHCs and the inconvenience of travelling multiple times to the vaccination center were key challenges for the caregivers. Caregivers mentioned that they would prefer fewer visits to session sites. A few caregivers mentioned that they would prefer their child was given multiple injections at one time as the infant would have to go through the experience only once instead of multiple times.

*“We travel a long way for the vaccine. So, if it is given once in 3–4 months, then it will be easier for us. Also, the child will have time to recover. It is easier for us also.”*

*“I feel that giving multiple vaccines at once is good. If we are giving vaccines every month, then it will be very difficult. We will have to go through the vaccination process every month.”*

Reaching migrant and tribal populations posed additional barriers due to tracking difficulties. These communities often moved frequently, complicating follow-ups. A state immunization officer (SIO) noted,

*“Tracking beneficiaries and follow-up is often difficult; it is tough to find previous records...vaccinators are sometimes hesitant to vaccinate a child from another catchment area.”*

**Figure 4:**
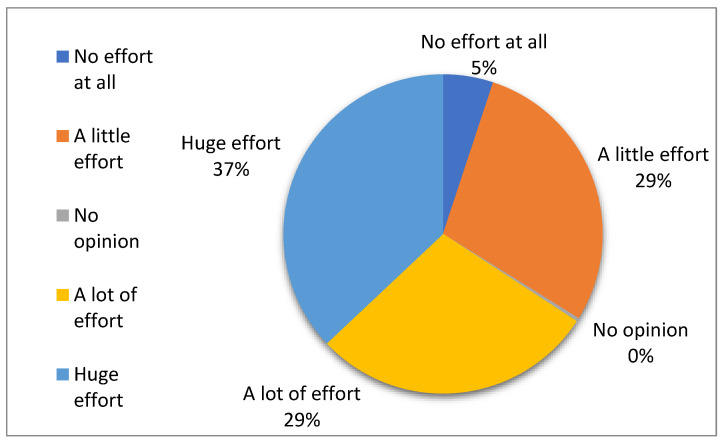
Caregivers’ burden/effort post-administration of multiple injectable vaccines in a single visit.

**Figure 5:**
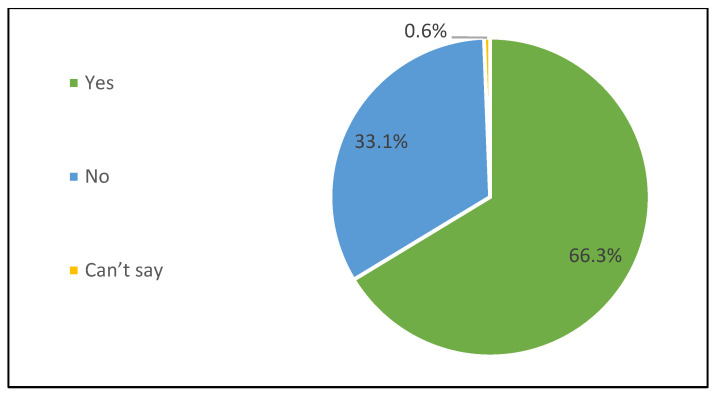
Caregivers’ perceived burden/effort post-vaccination: Taking care of beneficiary after multiple injectable vaccines interfered with other priorities.

According to the telephonic survey, caregivers emphasized the burden and effort post-vaccination. About 29% of the sample mentioned that they had to put in “a lot of effort” and 37% reported putting in “a huge effort” after the administration of multiple injectable vaccines in a single visit (Figure 4). Irrespective of the effort put in by the caregiver post-administration of multiple injectable vaccines, 66.3% reported that taking care of the beneficiary post-vaccination with multiple injectable vaccines interfered with other priorities during the day (Figure 5).

#### 3.2.2. Livelihood and Economic Constraints

For caregivers, particularly daily wage earners, attending vaccination sessions meant a loss of income. Medical officers (MOs) emphasized this challenge:

*“When the child cries, she must take care of the child...she is not earning money...they feel they are not gaining anything from it.”*

### 3.3. Perceptions, Knowledge, and Awareness

#### Caregivers’ Concerns About Pain and Side Effects

**Figure 6:**
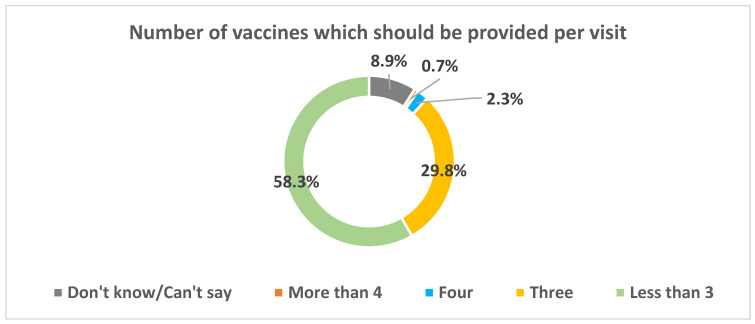
Caregivers’ perspective on multiple vaccine acceptance.

Caregivers expressed significant concerns about the physical discomfort of multiple injections. Fear of adverse events, such as swelling or fever, also discouraged vaccination uptake (Figure 6). One caregiver explained,

*“After the 9th-month vaccine, my baby had swelling...the fever was not subsiding. We feel there should be a gap between each vaccine.”*

Mothers, especially first timers, exhibited heightened anxiety, with most preferring fewer injections in one visit or spacing out doses. This was also noted by MOs:

*“Those with their first child remain a little conscious...everything is new for them, and it is difficult to console them.”*

The telephonic survey with caregivers suggested that majority of the caregivers felt that a maximum of three injectable vaccines should be provided in a single visit. Only 0.7% of the respondents agreed with providing more than four injectable vaccines per visit (Figure 6). A steep majority (85.4%) of the caregivers mentioned that the administration of more than four injectable vaccines to their ward in a single visit was unacceptable to them.

**Figure 7:**
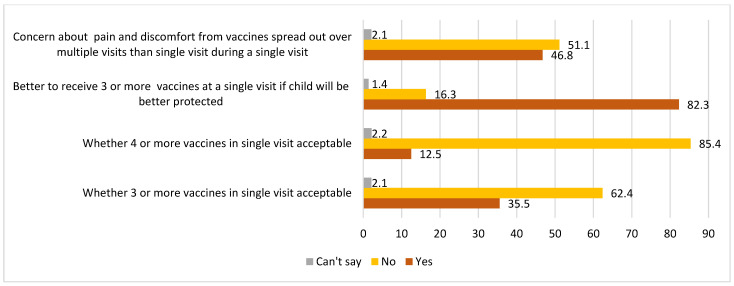
Caregivers’ perspectives on the concern about pain and number of injectable vaccines acceptable in a single visit.

The quantitative findings further reiterated the qualitative ones, with a majority of caregivers preferring multiple injectable vaccines to be spread over multiple visits rather than receiving multiple vaccines in a single visit (Figure 7).

### 3.4. Positive Indicators of Acceptability

Despite challenges, several respondents acknowledged the community’s gradual adaptability to new vaccines. ASHAs and MOs observed that repeated communication and the demonstration of vaccine benefits could mitigate resistance. As one caregiver put it,

*“If they keep calling us to inform and check about the (side) effects, it helps reduce fear.”*

Additionally, 82.3% caregivers agreed to receive three or more injectable vaccines in a single visit if their child would be “better protected” (Figure 7). This finding further highlights the role of the vaccinator and community mobilizer in reinforcing the benefits of vaccines being administered.

Furthermore, a preference for combining multiple vaccines into fewer sessions emerged among some caregivers. One mother remarked: “If vaccines are given together, it is easier for the child to recover and for us to manage”.

#### Operational Approaches for Inclusion of Additional Injectable Vaccine

The inclusion of an additional injectable vaccine under the Universal Immunization Program (UIP) was examined through operational perspectives, categorized under key themes: timeframe for administration, vaccine presentation, and system-level readiness. Insights were drawn from program managers, health workers, and caregivers.

### 3.5. Timeframe for Administration

There was consensus among program managers around introducing the vaccine between 16 to 24 months, aligning with reduced maternal antibody protection and increased child immunity from complementary feeding. As a state immunization officer (SIO) explained,

*“Children under 1 year are usually undernourished, and it’s better to give additional vaccines when they start eating complementary food.”*

Delaying introduction until 16–24 months was also suggested to leverage a period with fewer vaccines and less caregiver anxiety. An MO noted,

*“At 16–24 months, the child is stronger, and parents are less panicked.”*

Caregivers similarly felt that spacing out vaccines allowed children to recover between doses:

*“It’s better if there’s a gap... after 16 months, there’s no vaccine until 5 years.”*

**Figure 8:**
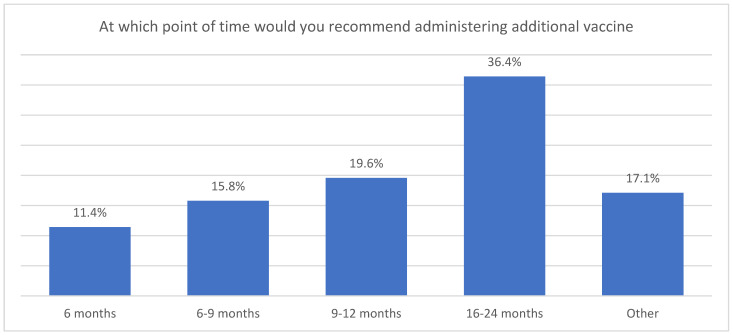
Caregivers’ recommendation for administering additional vaccine.

In the quantitative findings, too, more caregivers felt comfortable with introducing the additional vaccine at a later stage; 36.4% of the sample recommended administering the additional injectable vaccine between 16 and 24 months, while 19.5% recommended the 9-to-12-months touchpoint (Figure 8).

### 3.6. Vaccine Presentation

Stakeholders emphasized the need to balance caregiver acceptance and cost-effectiveness. Single-dose vials were preferred during the introductory phase to reduce wastage and reassure caregivers about vaccine freshness. A medical officer (MO) observed, “*The single dose is best... if you open a 10-dose vial and only four children are present, the wastage is more.*”

However, 5- or 10-dose vials were favored for routine use due to reduced costs and less biomedical waste. District immunization officers (DIOs) noted,

*“For economies of scale, 5- or 10-dose vials would be preferred.”*

A majority of health workers supported combining the new vaccine with existing ones, such as pentavalent or hexavalent formulations, to minimize the need for additional injections. An MO explained,

*“It is economical and reduces the number of visits, which is easier for working mothers.”*

Many respondents preferred oral vaccines due to higher caregiver acceptability and ease of administration. A DIO remarked,

*“It would be better if that could be an oral vaccine, like polio or rota.”*

### 3.7. System-Level Readiness

Service providers emphasized the importance of comprehensive training for all health workers, including auxiliary nurse midwives (ANMs) and accredited social health activists (ASHAs), on vaccine benefits, administration, and adverse event management. A DIO noted,

*“In 2017, we prepared ANMs and MOs on how to counsel parents about the MR vaccine... otherwise, parents resist.”*

ASHAs highlighted the need for detailed training to ensure they could effectively address community concerns. One ASHA shared,

*“Only if I know the full matter clearly can I explain and motivate them.”*

A strong focus on information, education, and communication (IEC) strategies was emphasized. MOs suggested leveraging local influencers, such as religious leaders, to dispel myths and improve uptake. An ANM emphasized, “Community awareness at a mass level, like polio campaigns, is critical”.

The role of ASHAs in mobilizing communities was highlighted as crucial, especially among migrant and underserved populations. Caregivers acknowledged their trust in ASHAs:

*“If ASHA sister explains, people feel confident about the vaccine.”*

## 4. Discussion

The findings of this study provide critical insights into the acceptability and operational challenges of introducing an additional injectable vaccine under the Universal Immunization Program (UIP). This discussion contextualizes these results within the framework of the existing literature to identify key alignments and implications for programmatic strategies.

The results highlight caregivers’ concerns about the pain and potential side effects associated with multiple injections, which align with findings from Idoko et al. and Tabana et al., who documented reluctance among caregivers due to similar fears [11,12]. In the present study, caregivers preferred fewer vaccination sessions visits, favoring combination vaccines to minimize inconvenience—a preference also identified in Dolan et al., where a single visit was deemed favorable for both caregivers and health workers [11]. Previous studies have demonstrated that combination vaccines, although more expensive, are cost effective and enhance compliance and the timeliness of vaccination. Combination vaccines offer to be a convenient and more acceptable approach that simplifies the administrative process for introducing an additional vaccine [13,14,15,16].

The present study suggested the role played by the vaccinators and community mobilizers contributing to the acceptance of more than three injectable vaccines in a single visit. This was in line with previous studies, which highlighted that trust in health workers, particularly ASHAs (community mobilizers), and positive recommendations by vaccinators significantly increased caregiver acceptance [17,18].

Geographical and resource-related barriers, such as difficult terrain, were identified as critical challenges. These findings align with the WHO’s observations on system readiness, which emphasizes the need for robust infrastructure to support vaccine introduction in resource-constrained settings [19,20]. Furthermore, the additional workload reported by health workers in this study mirrors the operational inefficiencies highlighted in a previous study that noted similar strains on health systems [18]. The need for comprehensive capacity building was consistently emphasized by stakeholders. Training health workers on vaccine administration, adverse event management, and community engagement was seen as vital, corroborating the findings of a study reporting that capacity-building efforts significantly improved vaccine uptake and community trust [21].

The preference for single-dose vials to multi-dose vials for injectable vaccine administration reported in the present study was in line with recommendations made in previous WHO position papers, which advocate minimizing vaccine wastage while addressing caregiver apprehensions [6]. The present study also highlights the importance of timing for vaccine administration, with stakeholders favoring introduction between 16 to 24 months. This aligns with the WHO guidelines recommending TCV administration at 9 months or 15–18 months, recognizing the benefits of aligning vaccination schedules with periods of lower maternal antibody protection and improved child immunity [6]. Additionally, it would also provide continued immunity following waning of the maternal immunity [22,23,24].

Furthermore, integrating new vaccines into existing formulations, such as pentavalent or hexavalent vaccines, can reduce the frequency of injections, thereby enhancing caregiver compliance and health worker efficiency. This strategy aligns with global evidence advocating for reduced injection frequencies to optimize programmatic outcomes [20,25].

This study has several limitations that should be considered while interpreting the findings. First, the study relied on self-reported data from caregivers and healthcare providers, which may be subject to recall bias and social desirability bias. Participants might have provided responses they perceived as more acceptable rather than their true opinions or experiences. Second, the study was conducted in select states with existing typhoid surveillance sites, which may not be fully representative of the diverse socio-cultural and geographical variations across India. The perspectives of stakeholders in other states with different immunization challenges and healthcare infrastructure may differ from the findings presented here. Another limitation pertains to the study design. While the mixed-methods approach provided comprehensive insights, the qualitative component relied on a purposive sampling strategy, which limits the generalizability of the findings. Additionally, the quantitative data collection was conducted through telephonic surveys, which excluded caregivers without access to mobile phones or those from marginalized communities, potentially introducing selection bias. Furthermore, the sample size for quantitative data collection was uneven across states, with some states being potentially underrepresented. This disparity could influence the overall findings and reduce the generalizability of the results at the national level.

Based on the findings, several key recommendations emerge to facilitate the successful introduction of an additional injectable vaccine under the Universal Immunization Programme (UIP). First, strategic communication and community engagement should be strengthened to address caregiver concerns regarding multiple injections in a single visit. Targeted information, education, and communication (IEC) campaigns, leveraging trusted healthcare workers such as accredited social health activists (ASHAs) and community leaders, can help dispel myths and enhance vaccine acceptance. Additionally, pre-vaccination counseling for caregivers should be emphasized to mitigate fears related to pain, side effects, and vaccine efficacy.

Second, vaccine presentation and scheduling strategies should be optimized to improve uptake and minimize operational challenges. Considering stakeholder preferences, introducing a combination vaccine or leveraging an oral formulation where feasible could reduce the number of injections per visit, improving caregiver acceptability.

Lastly, health system preparedness is critical for the effective rollout of an additional injectable vaccine. Comprehensive training programs for vaccinators, medical officers, and ASHAs should focus on vaccine administration, adverse event management, and caregiver counseling.

## 5. Conclusions

This novel study provides insight into the perspectives of program managers, vaccinators, community mobilizers, and caregivers regarding the introduction of an additional injectable vaccine in a single visit under the UIP. Given that the existing National Immunization Schedule has multiple injectable vaccines at each touchpoint, an AIV would put forth multiple challenges, as highlighted in the present study. Key points brought to the fore included the limited acceptability of more than three injectable vaccines in a single visit, the preference for a combination vaccine, and an inclination towards a lesser number of vaccination visits among caregivers.

The acceptability of an additional injectable vaccine in a single visit is influenced by multifaceted factors, including caregivers’ socioeconomic constraints, the age of the beneficiary, and contextual factors. Tailored interventions addressing these dimensions could enhance uptake and adherence under UIP.

In conclusion, the successful integration of an additional injectable vaccine requires a multi-pronged operational strategy, focusing on tailored vaccine presentations such as a combination vaccine (e.g., a hexavalent or pentavalent vaccine) or orally administered vaccine. An appropriate time window for the administration also came across as a key driver for acceptability.

## Figures and Tables

**Table 1:** National Immunization Schedule under UIP in India.

Age	Vaccines Given
Birth	Bacillus Calmette Guerin (BCG), Oral Polio Vaccine (OPV)-0 dose, Hepatitis B birth dose
6 Weeks	OPV-1, Pentavalent-1, Rotavirus Vaccine (RVV)-1, Fractional dose of Inactivated Polio Vaccine (fIPV)-1, Pneumococcal Conjugate Vaccine (PCV)-1
10 Weeks	OPV-2, Pentavalent-2, RVV-2
14 Weeks	OPV-3, Pentavalent-3, fIPV-2, RVV-3, PCV-2
9–12 Months	Measles & Rubella (MR)-1, JE-1 **, PCV-Booster
16–24 Months	MR-2, JE-2 **, Diphtheria, Pertussis, and Tetanus (DPT)-Booster-1, OPV–Booster
5–6 Years	DPT-Booster-2
10 Years	Tetanus and Adult Diphtheria (Td)
16 Years	Td
Pregnant Mother	Td-1, Td-2, or Td-Booster ***

** Administered in select endemic states; *** if pregnancy occur within 3 years of last pregnancy and 2 Td doses were received.

**Table 2:** State-wise sample for quantitative data collection.

State	Final Sample	% of Sample
Himachal Pradesh	278	24.4
Delhi	102	8.9
Bihar	50	4.4
Assam	204	17.9
West Bengal	145	12.7
Maharashtra	119	10.4
Andhra Pradesh	155	13.6
Tamil Nadu	87	7.6
Total	1140	100.0

**Table 3:** Qualitative sampling by respondent category.

Method	Stakeholder	Total
IDI	SEPIO	8
	DIO	8
	MO	16
	ANM	32
	ASHA	32
FGD	Caregivers	16
Total		112

## Data Availability

The anonymized datasets used and/or analyzed during the current study are available from the corresponding author on reasonable request.
